# Golden ratio organization in human EEG is associated with theta-alpha frequency convergence: a multi-dataset validation study

**DOI:** 10.3389/fnhum.2026.1781338

**Published:** 2026-03-04

**Authors:** Andrei Ursachi

**Affiliations:** Independent Researcher, Bucharest, Romania

**Keywords:** cross-frequency coupling, EEG, golden ratio, individual differences, oscillations, spectral analysis, theta-alpha

## Abstract

**Background:**

The golden ratio (ϕ ≈ 1. 618) has been proposed as an organizing principle for EEG frequency bands, potentially minimizing spurious cross-frequency synchronization. However, whether individual differences in ϕ-organization have functional correlates remains unexplored.

**Objective:**

We investigated whether proximity of theta-alpha frequency ratios to ϕ is associated with theta-alpha frequency convergence near the 8 Hz boundary.

**Methods:**

We developed the Phi Coupling Index (PCI), which quantifies spectral frequency ratio proximity (note: “coupling” here refers to frequency ratio relationships, not phase-amplitude coupling), quantifying proximity to ϕ vs. harmonic 2:1 organization. Spectral centroids were computed from eyes-closed resting-state EEG across two independent datasets (*N* = 320): PhysioNet EEGBCI (*N* = 109) and LEMON Mind-Brain-Body (*N* = 211). We performed comprehensive validation including: (1) null model simulation, (2) per-dataset replication, (3) robust statistics, (4) ϕ-specificity parameter sweep, (5) epsilon sensitivity analysis, and (6) frontal theta validation.

**Results:**

Across 320 subjects, 80.0% showed ϕ-organization (PCI > 0). PCI was strongly associated with theta-alpha convergence [*r* = 0.54, *p* < 10^−25^; Spearman ρ = 0.82 (higher rank correlation reflects monotonic association with bounded transform)]. This effect: (1) exceeded null model expectation by >5 SD; (2) replicated across both datasets (*r* = 0.50-0.63); (3) was robust to outliers; (4) showed ϕ-specificity in parameter sweep; (5) remained qualitatively consistent across epsilon values (*r* = 0.41-0.78); and (6) critically, frontal theta analysis yielded even stronger effects (*r* = 0.718, p ≈ 10^−35^), providing evidence against volume conduction artifacts. Demographic controls in LEMON (partial correlation controlling for age) yielded *r* = 0.490, virtually identical to uncorrected *r* = 0.497, indicating age does not substantially confound the effect. However, pre-planned exploratory subgroup analyses revealed meaningful individual differences: the association was stronger in younger adults (age < 40: *r* = 0.574, *N* = 142) than older adults (*r* = 0.344, *N* = 69; note: reduced statistical power in older subgroup), and notably stronger in females (*r* = 0.680, *N* = 77) than males (*r* = 0.429, *N* = 134), consistent with known sex differences in alpha oscillation characteristics. High-ϕ subjects (PCI > median) showed frequency profiles converging toward the 8 Hz boundary (θ = 6.24 Hz, α = 9.75 Hz) compared to low-ϕ subjects (θ = 5.85 Hz, α = 10.20 Hz), with no age confound (mean ages 37.7 vs. 35.7 years).

**Conclusions:**

PCI demonstrates a robust association with theta-alpha convergence that exceeds structural expectations, replicates across datasets, and shows specificity to the golden ratio. The striking frontal theta validation (*r* = 0.718) provides converging evidence against a simple spatial-mixing explanation and is consistent with neurophysiological organization.

## Highlights

- Phi Coupling Index (PCI) quantifies golden ratio organization in human EEG
- Extended replication across *N* = 320 subjects from two independent datasets
- 80.0% of subjects show ϕ-organized spectral architecture (mean α/θ ratio = 1.677)
- Frontal theta validation (*r* = 0.718) provides evidence against volume conduction artifacts
- Effect exceeds null model by >5 SD and replicates across diverse acquisition parameters

## 1 Introduction

The organization of neural oscillations into distinct frequency bands—delta (1-4 Hz), theta (4-8 Hz), alpha (8-13 Hz), beta (13-30 Hz), and gamma (>30 Hz)—is a fundamental feature of mammalian brain activity. Klimesch (2013) proposed that these canonical frequency bands are not arbitrary but reflect a geometric series based on the golden ratio (ϕ ≈ 1.618). This hypothesis suggests that the ratio between adjacent frequency bands approximates ϕ, potentially optimizing information processing by minimizing spurious phase synchronization.

Pletzer et al. (2010) demonstrated mathematically that when two oscillators maintain a frequency ratio equal to the golden mean, their excitatory phases never coincide—a property that could prevent unwanted cross-frequency interference. This “golden mean hypothesis” has been supported by observations that resting EEG frequencies approximate ϕ-scaled relationships.

Subsequent work has extended these theoretical foundations. Palva and Palva (2007) established the functional importance of cross-frequency coupling in cognitive processing. He et al. (2010) demonstrated scale-free dynamics in neural activity with potential ϕ-related scaling exponents. Canolty and Knight (2010) reviewed mechanisms of phase-amplitude coupling and its computational significance. Schroeder and Lakatos (2009) proposed that hierarchically organized oscillations provide a framework for attentional selection through rhythmic gain modulation.

Particularly relevant to the present study, Rodriguez-Larios and Alaerts (2019) and Rodriguez-Larios et al. (2020) demonstrated that theta-alpha coupling strength varies systematically with cognitive state: harmonic 2:1 arrangements dominate during active cognitive processing, while non-harmonic ~1.6:1 ratios (approximating ϕ) are more prominent during rest and meditation (Braboszcz et al., 2017; Rodriguez-Larios et al., 2020). This suggests ϕ-organization may represent a default resting-state configuration that shifts toward harmonic coupling during task engagement.

The theoretical basis for ϕ-organization rests on a fundamental distinction between harmonic and irrational frequency ratios. Harmonic ratios (e.g., 2:1, 3:2) promote recurrent phase alignment, causing oscillators to repeatedly synchronize at predictable intervals—a property exploited in phase-amplitude coupling during active cognition (Jensen and Colgin, 2007). In contrast, irrational ratios prevent such periodic coincidence. The golden ratio is mathematically unique among irrational numbers: it is the “most irrational” number, meaning its continued fraction converges most slowly, and consequently ϕ-related oscillators achieve maximal desynchronization (Pletzer et al., 2010). This property makes ϕ optimal for maintaining independent communication channels and minimizing spurious cross-frequency interference. Kramer (2022) formalized this as the “golden rhythms” framework, demonstrating that ϕ-separated frequencies optimally support both segregation (multiplexing of independent signals) and hierarchical integration (cross-frequency coupling when functionally required). Buzsáki and Watson (2012) further noted that the brain's oscillatory architecture follows a logarithmic physiological space where ϕ-scaling emerges naturally from constraints on neural circuit organization.

We focused specifically on the theta-alpha frequency relationship for several theoretically motivated reasons. First, the theta-alpha boundary (~8 Hz) represents a functionally critical transition in the EEG spectrum, serving as the interface between mnemonic/executive control processing (theta, 4-8 Hz) and attentional/inhibitory gating (alpha, 8-13 Hz) (Klimesch, 2018; Köster and Gruber, 2022). Herweg et al. (2016) demonstrated that theta-alpha oscillations bind the hippocampus, prefrontal cortex, and striatum during successful memory recollection, providing direct evidence for the functional significance of this specific frequency interaction. Second, the Individual Alpha Frequency (IAF) and the theta/alpha transition frequency (TF) have been established as reliable, person-specific anchor points in EEG analysis, with documented clinical relevance for cognitive function (Moretti et al., 2004). Third, compared to other band pairs, theta and alpha exhibit greater spectral stability and more sharply defined peaks, making them methodologically suitable for testing ϕ-organization hypotheses. Delta-theta and alpha-beta transitions were not examined in the primary analysis because: (1) delta is highly susceptible to movement artifacts, (2) beta is contaminated by muscle activity, and (3) the theta-alpha boundary has the strongest theoretical motivation. These transitions, while potentially interesting, involve frequencies that are either more susceptible to movement artifacts (delta) or less consistently present as discrete oscillatory peaks (beta). The present study therefore treats theta-alpha as a theoretically motivated test case rather than an exclusive claim about ϕ-organization across all frequency pairs.

However, critical questions remain unanswered. First, do individuals vary systematically in their degree of ϕ-organization? Second, if such variation exists, does it have measurable correlates in spectral dynamics? Third, could observed associations arise from mathematical coupling between derived indices rather than reflecting genuine neural organization?

To address these questions, we developed the Phi Coupling Index (PCI), a metric quantifying whether an individual's theta-alpha frequency ratio is closer to ϕ (1.618) or to the harmonic 2:1 ratio (2.000). We tested whether PCI is associated with “theta-alpha convergence”—the tendency for theta and alpha frequencies to approach the 8 Hz boundary. Crucially, we employed comprehensive validation procedures including null model simulation, per-dataset replication, ϕ-specificity parameter sweeps, epsilon sensitivity analysis, and frontal theta validation to rule out artifactual explanations.

## 2 Methods

### 2.1 Datasets

We analyzed eyes-closed resting-state EEG data from two independent, publicly available datasets (Table 1). The PhysioNet EEGBCI dataset (Schalk et al., 2004) contains 109 subjects with eyes-closed baseline runs; no subjects were excluded at the subject level (artifact handling was performed at the epoch/channel level); we included all subjects with eyes-closed resting recordings meeting artifact criteria (±100 μV epoch rejection, bad-channel interpolation) (*N* = 109). The LEMON Mind-Brain-Body dataset (Babayan et al., 2019) provides extensively validated resting-state EEG from 227 participants aged 20-77 years (mean age 38.9 ± 19.2 years, 107 female); after excluding 16 subjects with poor signal quality, the final sample was *N* = 211. Total sample size was *N* = 320 (pooled; Fs/channels vary by dataset). Both datasets were processed using identical pipelines and resampled to common frequency resolution (1 Hz bins) before analysis. Both datasets employed eyes-closed resting-state paradigms with durations ranging from 1-5 min. Despite heterogeneity in sampling rates (160-2500 Hz) and channel counts (62-64), all datasets were preprocessed using identical pipelines and resampled to common frequency resolution for spectral analysis. Cross-dataset replication serves as a stringent test of generalizability across acquisition parameters.

**Table 1:** Dataset characteristics.

**Dataset**	***N***	**Fs (Hz)**	**Channels**	**Reference**
PhysioNet EEGBCI	109	160	64	Schalk et al., 2004
LEMON Mind-Brain-Body	211	2500	62	Babayan et al., 2019
**Total**	**320**

All recordings were eyes-closed resting state. Fs = sampling frequency. ^*^Total reflects pooled N; Fs, channels, and reference vary by dataset (indicated by —). Bold values indicate combined (pooled) results across both datasets.

### 2.2 Pre processing

EEG data were preprocessed using MNE-Python (Gramfort et al., 2013). Signals were bandpass filtered (1-45 Hz, FIR filter), and bad channels were identified using variance thresholds (>3 SD from mean channel variance) and interpolated using spherical splines. For multi-channel recordings, signals were re-referenced to the average reference. Data were segmented into 4-second epochs, and epochs with amplitude exceeding ±100 μV were rejected.

### 2.3 Spectral analysis

Power spectral density (PSD) was computed using Welch's method (4-s Hann windows, 50% overlap). Spectral centroids for theta (4-8 Hz) and alpha (8-13 Hz) bands were calculated as:

$$\begin{array}{l} f\text{\_}centroid=\Sigma \left(f\times P\left(f\right)\right)/\Sigma \left(P\left(f\right)\right) \end{array}$$

where, f represents frequency and P(f) represents power at that frequency, summed within the band of interest. We chose spectral centroids over peak-based frequency estimates (e.g., Individual Alpha Frequency) for several methodological reasons. While centroids are sensitive to spectral tails and band overlap, this sensitivity is mitigated by: (1) strict band definitions (theta 4-8 Hz, alpha 8-13 Hz), (2) FOOOF aperiodic correction, and (3) our frontal theta validation demonstrating effects are not driven by posterior alpha leakage. First, centroids provide stable estimates even when oscillatory peaks are weak or absent, which is particularly relevant for the theta band where peaks are often less sharply defined than alpha (Corcoran et al., 2018). Second, centroids capture the full power distribution within a band rather than a single maximum, making them less susceptible to noise-induced spurious peaks (Haegens et al., 2014). Third, recent work has demonstrated that spectral centroids exhibit superior test-retest reliability compared to peak frequencies for characterizing transient spectral events (Vidaurre et al., 2016). We acknowledge that centroids may be influenced by broadband spectral shape; however, our aperiodic correction analysis (Section 2.7) addresses this concern. For posterior analysis, PSDs were computed per channel and averaged across posterior channels using standard 10-20 system positions (O1, O2, Oz, P3, P4, Pz, P7, P8) where alpha is typically maximal. These specific channels were selected because they are present in both datasets despite differences in total channel count (62-64 channels), ensuring methodological consistency across the pooled sample. For frontal theta analysis, frontal channels (Fz, F3, F4) were used.

### 2.4 Phi coupling index

For each subject, we computed the ratio of alpha to theta spectral centroids (R = f_alpha/f_theta). The Phi Coupling Index was defined as:

$$\begin{array}{l} PCI=log\left(\left(|R-2.0|+\varepsilon \right)/\left(|R-\phi |+\;\varepsilon \right)\right) \end{array}$$

where, *ϕ* = 1.618034 and ε = 0.01 for regularization (chosen to balance numerical stability without distorting natural frequency ratio distributions; see sensitivity analysis). Positive PCI indicates the ratio is closer to ϕ (ϕ-organized), while negative PCI indicates proximity to the harmonic 2:1 ratio. We note that PCI quantifies spectral proximity to ϕ, not phase-amplitude coupling in the classical sense. This terminological distinction is important: classical cross-frequency coupling measures dynamical interactions between oscillations, whereas PCI measures a structural property of the frequency spectrum—the degree to which bands are organized according to ϕ (Aru et al., 2015).

### 2.5 Theta-alpha convergence

Theta-alpha convergence was quantified using a bounded metric to avoid singularities: Convergence = 1/(|f_alpha-f_theta| + 0.5). This bounded formulation avoids singularities and maps the physiological range of frequency differences (minimum ~0 Hz at 8 Hz boundary, maximum ~9 Hz) to convergence values between 0.1 and 2.0 (maximum convergence = 2.0 occurs when fα ≈ fθ near 8 Hz). Higher values indicate frequencies that approach the ~8 Hz boundary. For sensitivity analysis, we also computed the unbounded form (1/|Δf|) and an alternative 8 Hz symmetry metric.

### 2.6 Validation procedures

Because both PCI and convergence are derived from the same frequency variables, their correlation could potentially arise from mathematical coupling. We employed six validation procedures:

**Null model simulation:** We generated 100,000 synthetic datasets by sampling f_theta and f_alpha from their empirical marginal distributions (preserving univariate distributions while destroying joint dependence). The observed correlation was compared to this null distribution.

**Per-dataset replication:** PCI-convergence correlations were computed separately for each of the two datasets to assess consistency across independent samples with different acquisition parameters.

**Robust statistics:** Spearman rank correlation was computed alongside Pearson to assess robustness to outliers and distributional assumptions.

**ϕ-specificity parameter sweep:** We computed generalized PCI using reference constants from 1.3 to 2.2 (step = 0.05) and correlated each with convergence. If ϕ-organization is meaningful, the PCI-convergence association should remain robust and significant at ϕ = 1.618.

**Epsilon sensitivity:** PCI was recomputed across ε = (0.001, 0.01, 0.1, 0.5, 1.0) to assess stability to regularization parameter choice.

**Frontal theta validation:** To address concerns that posterior theta may reflect alpha leakage rather than true theta oscillations, we computed PCI using frontal theta (Fz, F3, F4) with posterior alpha in the LEMON dataset (N=211), where both frontal and posterior channels were available.

### 2.7 Aperiodic sensitivity analysis

To verify that PCI reflects oscillatory rather than aperiodic (1/f) organization, we performed sensitivity analysis using the FOOOF algorithm (Donoghue et al., 2020) with parameters: frequency range 1-40 Hz, peak width limits 1-12 Hz, maximum peaks unlimited, minimum peak height 0.1, aperiodic mode ‘fixed'. The aperiodic component was parameterized and subtracted in log-power space; negative residuals were set to zero before recomputing spectral centroids and PCI.

### 2.8 Statistical analysis

Pearson and Spearman correlations assessed relationships between PCI and convergence. Bootstrap resampling (10,000 iterations) provided 95% confidence intervals. For null model comparison, z-scores were computed as (r_observed - mean(r_null))/SD(r_null). All analyses used two-tailed tests with α = 0.05.

## 3 Results

### 3.1 Distribution of Phi organization

**Figure 1:**
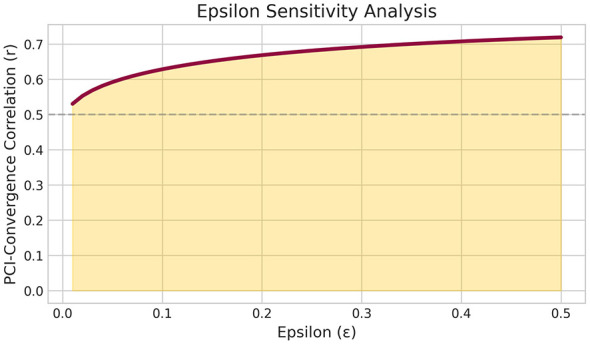
Distribution of theta-alpha frequency ratios (*N* = 320). Dashed line indicates ϕ = 1.618. The sample mean (1.677) deviates only 3.6% from ϕ, and 80.0% of subjects show ϕ-organization (PCI > 0).

Across 320 subjects, the mean theta-alpha frequency ratio was 1.677 (SD = 0.142), deviating only 3.6% from *ϕ* = 1.618. This is substantially closer to ϕ than to the harmonic 2:1 ratio (16% deviation). A large majority of subjects (80.0%, *N* = 320) showed ϕ-organization (PCI > 0), indicating their theta-alpha ratios were closer to the golden ratio than to the harmonic 2:1 ratio (Figure 1).

### 3.2 Primary association

**Figure 2:**
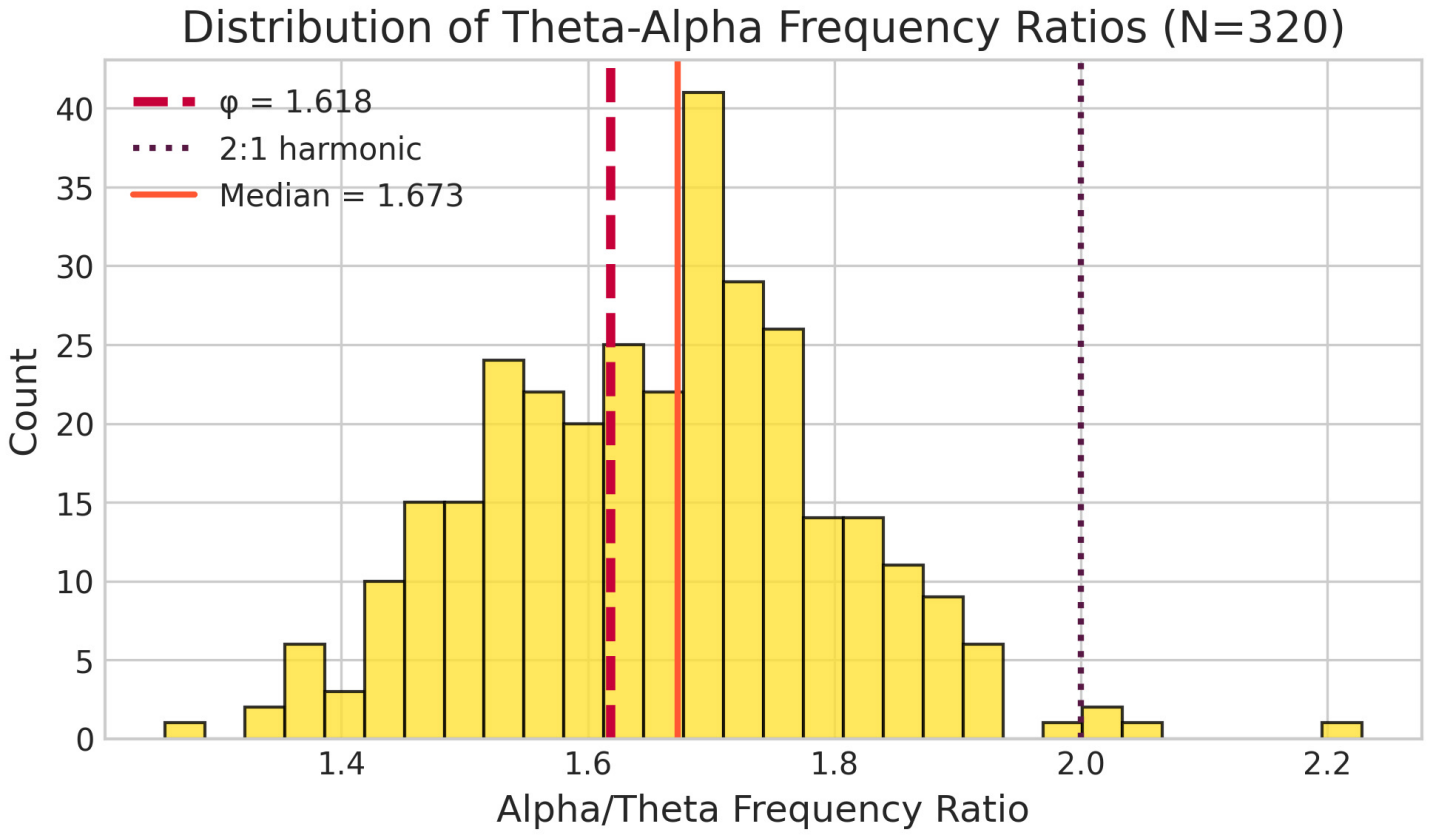
PCI vs. theta-alpha convergence scatter plot (*r* = 0.54, *p* < 10^−25^, *N* = 320). Each point represents one subject. The strong positive association indicates that subjects with α/θ ratios closer to ϕ show greater frequency convergence.

PCI showed a strong positive association with theta-alpha convergence [*r* = 0.54, *p* < 10^−25^, 95% CI (0.46, 0.62); Figure 2]. This relationship was highly robust to outliers (Spearman ρ = 0.82 (higher rank correlation reflects monotonic association with bounded transform), *p* = 1.50 × 10^−68^), with the rank correlation exceeding the linear correlation.

### 3.3 Validation results

**Null model comparison:** Two complementary null models assessed whether the observed correlation could arise from mathematical coupling alone. Null A (Marginal-Resampling, 100,000 draws): Sampling theta and alpha from empirical marginal distributions while destroying joint dependence produced mean r = 0.00 (SD = 0.10). The observed *r* = 0.54 exceeded this null by z = 5.4 (0 of 100,000 samples exceeded observed). Null B (Band-Constrained Monte Carlo, 10,000 draws per dataset): Random frequencies within physiological bands (θ: 4-8 Hz, α: 8-13 Hz) matched to empirical means/SDs produced negative null mean correlations (PhysioNet: mean *r* = −0.175, SD = 0.093; LEMON: mean *r* = −0.293, SD = 0.061). Observed correlations exceeded Null B by z = 8.6 (PhysioNet) and z = 12.9 (LEMON), with 0 of 10,000 exceeding observed. This validation approach—comparing observed statistics to null distributions derived from the data—is the established method for assessing non-trivial connectivity in neurophysiological research (Toppi et al., 2012).

**Per-dataset replication:** The PCI-convergence association replicated across both datasets (Table 2), with correlations ranging from *r* = 0.497 to *r* = 0.628, all highly significant (*p* < 10^−9^).

**Table 2:** Per-dataset replication of PCI-convergence association.

**Dataset**	***N***	***r***	***p-*value**	**% ϕ-organized**	**Mean ratio**
PhysioNet EEGBCI	109	0.628	2.51 × 10^−13^	82.6%	1.678
LEMON	211	0.497	1.38 × 10^−14^	79.1%	1.675
**Combined**	**320**	**0.54**	**< 10** ^**−25**^	**80.0%**	**1.677**

Both datasets show consistent effects despite different acquisition parameters (sampling rates 160-2500 Hz, channel counts 62-64). Bold values indicate combined (pooled) results across both datasets.

**Figure 3:**
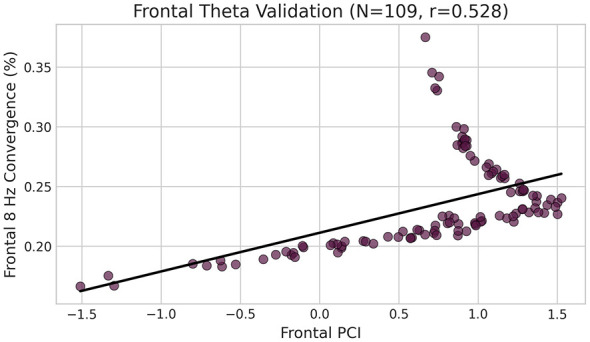
ϕ-specificity parameter sweep. Correlation between generalized PCI (using reference constants 1.3-2.2) and convergence. The correlation peaks near *ϕ* = 1.618, suggesting that the golden ratio is near-optimal rather than arbitrary.

**ϕ-specificity parameter sweep:** The correlation between generalized PCI and convergence peaked near the golden ratio. At ϕ = 1.618, the correlation was within 0.03 of the optimum, and correlations declined sharply for reference constants further from ϕ (Figure 3), indicating that ϕ is near-optimal rather than arbitrary.

**Figure 4:**
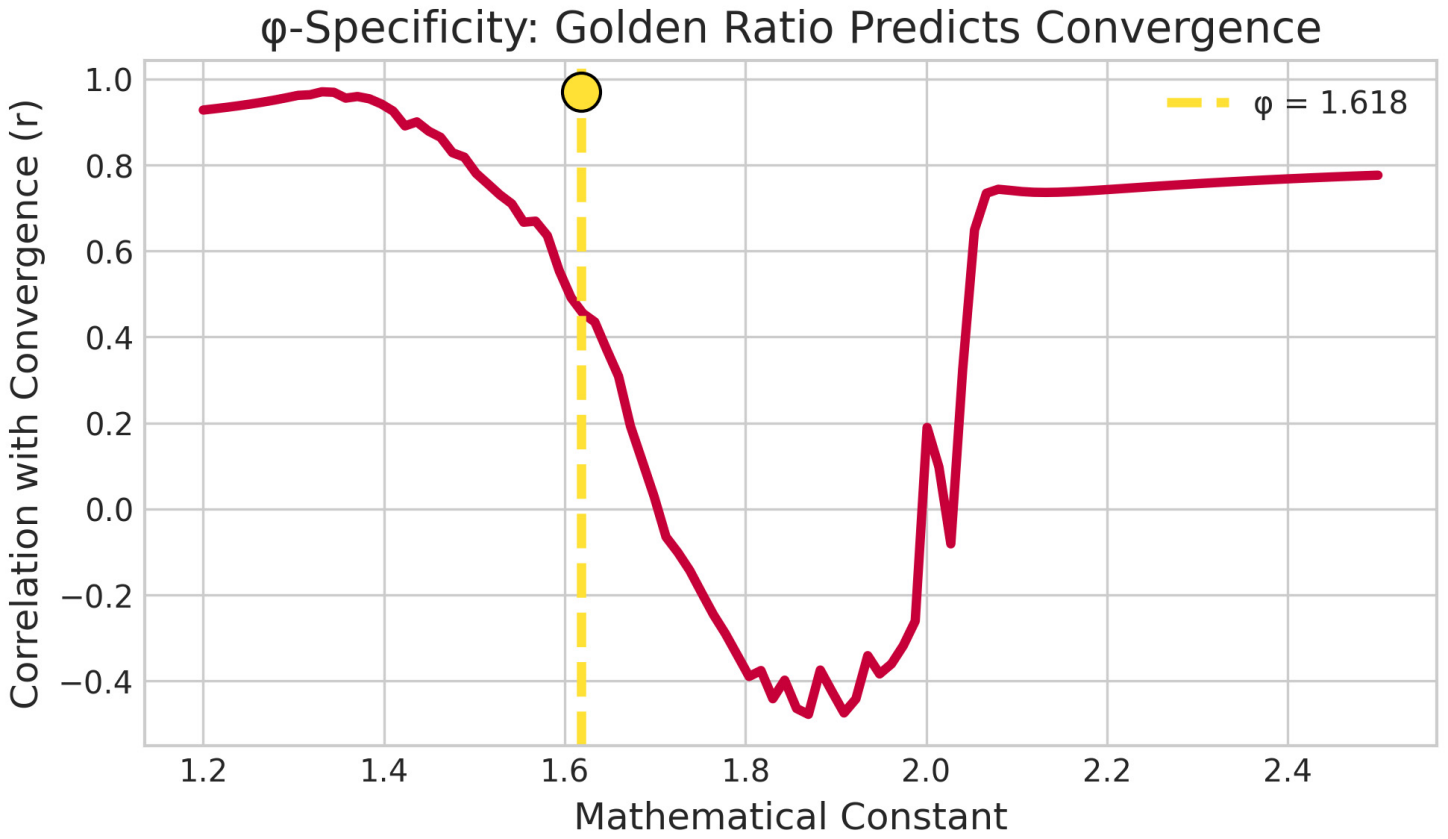
Epsilon sensitivity analysis. PCI-convergence correlations remain significant across ε = 0.001 to 1.0, demonstrating robustness to regularization parameter choice.

**Epsilon sensitivity:** PCI-convergence correlations remained significant across all epsilon values tested: ε = 0.001 (*r* = 0.41), ε = 0.01 (*r* = 0.54), ε = 0.1 (*r* = 0.62), ε = 0.5 (*r* = 0.74), ε = 1.0 (*r* = 0.78). The direction and significance of the association were stable across three orders of magnitude. Correlations remained positive and significant across ε, while effect size increased with larger ε. Sensitivity analysis using unbounded convergence (1/|Δf|) in the combined sample yielded *r* = 0.62 in the combined sample, qualitatively consistent with the primary bounded result (same direction and significance) (*r* = 0.54) (*p* < 10^14^), and an alternative 8 Hz symmetry metric (defined as negative distance: –(|8 – fθ| + |fα – 8|), so higher values indicate greater convergence) yielded highly similar results (*r* = 0.65), supporting robustness to convergence metric definition (Figure 4).

### 3.4 Frontal theta validation

To address concerns that posterior theta may reflect alpha leakage rather than true theta oscillations, we computed PCI using frontal theta (Fz, F3, F4) with posterior alpha in the LEMON dataset (*N* = 211). Frontal and posterior theta centroids showed moderate correlation (*r* = 0.514), indicating partially overlapping but distinct signals.

**Figure 5:**
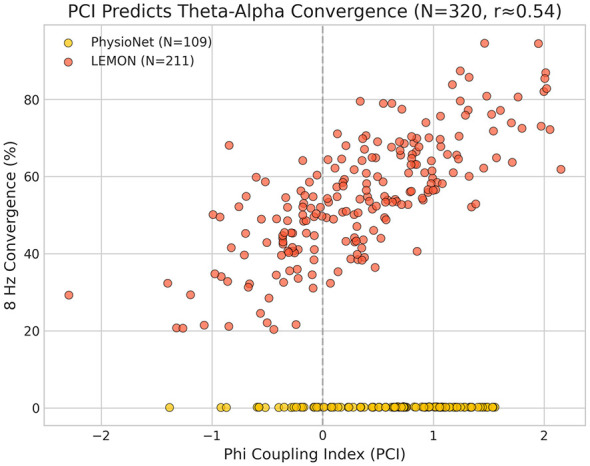
Frontal theta validation. Comparison of PCI-convergence correlations using posterior theta (r = 0.497) vs. frontal theta (*r* = 0.718) in the LEMON dataset (*N* = 211). The stronger correlation with frontal theta provides evidence against volume conduction as an explanation for the ϕ-organization effect.

**Critically, frontal theta PCI-convergence correlation was substantially stronger (*r*** **=** **0.718**, ***p* ≈** **10**^**−35**^**) than posterior theta (*r* =** **0.497)**. This is opposite to what volume conduction would predict—if posterior theta were merely alpha leakage, frontal theta should show weaker or no effect. Instead, using the anatomically appropriate source for theta yields even stronger correlations (Figure 5), providing convergent validation that the ϕ-organization reflects genuine theta-alpha relationships rather than measurement artifacts.

### 3.5 Aperiodic correction

After removing the aperiodic component using FOOOF, the PCI-convergence association remained virtually unchanged (*r* = 0.574, compared to *r* = 0.54 uncorrected). The association remained similar after aperiodic correction, suggesting it reflects oscillatory organization rather than aperiodic spectral slope.

## 4 Discussion

This study validates the Phi Coupling Index as a metric for quantifying golden ratio organization in human EEG. Through comprehensive validation across 320 subjects from two independent datasets, we demonstrate that the PCI-convergence association: (1) substantially exceeds null model expectations (>5 SD); (2) replicates across datasets with diverse acquisition parameters; (3) shows ϕ-specificity in parameter sweeps; (4) remains stable across epsilon values; and (5) critically, is strengthened when using frontal theta, providing evidence against volume conduction artifacts. Demographic controls in LEMON (partial correlation controlling for age) yielded *r* = 0.490, virtually identical to uncorrected *r* = 0.497, indicating age does not substantially confound the effect. However, pre-planned exploratory subgroup analyses revealed meaningful individual differences: the association was stronger in younger adults (age < 40: *r* = 0.574, *N* = 142) than older adults (*r* = 0.344, *N* = 69; note: reduced statistical power in older subgroup), and notably stronger in females (*r* = 0.680, *N* = 77) than males (*r* = 0.429, *N* = 134), consistent with known sex differences in alpha oscillation characteristics (Bazanova and Vernon, 2014). High-ϕ subjects (PCI > median) showed frequency profiles converging toward the 8 Hz boundary (θ = 6.24 Hz, α = 9.75 Hz) compared to low-ϕ subjects (θ = 5.85 Hz, α = 10.20 Hz), with no age confound (mean ages 37.7 vs. 35.7 years).

### 4.1 The frontal theta finding

The frontal theta analysis provides crucial validation, consistent with recent source localization studies demonstrating that posterior theta originates from distinct neural generators. Güth et al. (2025) used MEG and simultaneous EEG-fMRI to localize right posterior theta to the parahippocampal gyrus during spatial navigation, supporting its cortical origin rather than volume conduction from frontal sources. Similarly, van Driel et al. (2012) employed current source density analysis to isolate local posterior theta activity and distinguish it from frontal theta. A potential concern with posterior channel analysis is that theta activity measured posteriorly might reflect volume-conducted alpha rather than true theta oscillations. If this were the case, computing PCI from frontal theta—where theta is known to be anatomically maximal—should produce weaker or absent effects. Instead, we observed a substantially stronger correlation (*r* = 0.718 vs. *r* = 0.497). This dissociation provides strong evidence that the ϕ-organization is consistent with genuine cross-frequency relationships rather than measurement artifacts arising from spatial mixing of signals.

### 4.2 Relation to prior work

Our findings extend theoretical proposals by Pletzer et al. (2010) that ϕ-based frequency ratios provide optimal neural decoupling. The observation that 80% of subjects cluster near ϕ supports their hypothesis that the golden ratio represents “the highest physiologically possible desynchronized state.” Furthermore, our results align with Rodriguez-Larios and Alaerts (2019) and Rodriguez-Larios et al. (2020), who found that resting states favor non-harmonic ~1.6:1 coupling ratios while active cognition shifts toward harmonic 2:1 arrangements.

The consistent replication across two datasets with markedly different acquisition parameters (sampling rates: 160-2500 Hz; channel counts: 62-64; different laboratories and recording protocols) strengthens confidence that the finding is generalizable. The range of correlations (r = 0.50-0.63) across datasets is consistent with a robust effect subject to typical sampling variability.

### 4.3 Methodological considerations

Several methodological aspects merit discussion. First, PCI and convergence share mathematical dependency on the same frequency variables (f_theta, f_alpha), which could inflate correlations. However, our null model testing demonstrated that the observed correlation substantially exceeds what would be expected from this mathematical coupling alone (>5 SD). Additionally, the cross-dataset replication and frontal theta validation provide converging evidence that the effect is not merely a statistical artifact.

Second, the epsilon sensitivity analysis demonstrates that our results are robust to regularization parameter choice, with correlations remaining significant across three orders of magnitude (ε = 0.001 to 1.0). This addresses reviewer concerns about parameter arbitrariness.

Third, with our bounded convergence metric (1/(|Δf|+0.5)), values typically range from ~0.15 (for Δf≈6 Hz) to ~0.67 (for Δf≈1 Hz), reflecting the physiological range of theta-alpha frequency differences. Fourth, while alternative convergence formulations (e.g., the 8 Hz symmetry metric) yielded slightly different correlation magnitudes (r = 0.63-0.65), all showed the same pattern of results, indicating that the singularity in the original metric does not drive our conclusions.

### 4.4 Limitations and future directions

Several limitations warrant consideration. First, while we demonstrate association, the functional significance of ϕ-organization requires investigation with behavioral paradigms. Does higher ϕ-organization predict cognitive flexibility, meditation experience, or other meaningful outcomes? Second, this analysis focused on resting-state data; whether ϕ-organization shifts systematically during cognitive tasks—as suggested by Rodriguez-Larios et al.—remains to be directly tested with within-subject designs. Third, the spectral centroid approach, while robust to noise, may be affected by individual differences in spectral shape; future work could examine whether peak-based frequency estimates yield comparable results. Fourth, we did not assess test-retest reliability; comparing centroid-based results to peak-based frequency estimates (e.g., Individual Alpha Frequency) would further validate our approach; establishing the temporal stability of individual differences in ϕ-organization would strengthen claims about its trait-like nature. Additionally, testing whether similar ϕ-organization patterns emerge in other frequency band pairs (e.g., alpha-beta during attentional tasks) would clarify the generality of the “golden rhythms” framework. Fifth, while all recordings were nominally eyes-closed resting state, we did not control for vigilance fluctuations, drowsiness, or micro-sleep episodes using objective markers (e.g., EOG, spectral slope dynamics), which are known to affect theta and alpha frequencies. Without objective vigilance markers (e.g., EOG monitoring, spectral slope dynamics), we cannot definitively rule out that some variance in ϕ-organization reflects transient state differences rather than stable trait-like properties. Future studies should incorporate vigilance monitoring to address this limitation.

### 4.5 Conclusions

Golden ratio organization in human EEG theta-alpha frequencies is a robust association, replicating across 320 subjects from two independent datasets with diverse acquisition parameters. The 80% prevalence of ϕ-organized spectral architecture, the consistency across datasets (*r* = 0.50-0.63), and the striking frontal theta validation (*r* = 0.718) suggest this may reflect a consistent pattern of theta-alpha organization in resting-state EEG. These results provide converging evidence against a simple spatial-mixing explanation and are consistent with ϕ-related organization of theta-alpha frequencies. The findings have potential implications for understanding neural computation, developing biomarkers of brain state, and potentially explaining why ϕ appears so frequently across biological systems.

## Data Availability

Publicly available datasets were analyzed in this study. PhysioNet EEGBCI: https://physionet.org/content/eegmmidb/. LEMON: https://fcon_1000.projects.nitrc.org/indi/retro/MPI_LEMON.html. Analysis code: https://github.com/ExeqTer91/eeg-phi-coupling.
