# Supplementary material for: Golden ratio organization in human EEG is associated with theta-alpha frequency convergence: a multi-dataset validation study
Source: Front Hum Neurosci. 2026 Mar 4;20:1781338. doi: 10.3389/fnhum.2026.1781338 (PMC12996120; doi:10.3389/fnhum.2026.1781338)
Supplement: Supplementary file 2 [file Data_Sheet_1.pdf]

Supplementary Materials

Golden Ratio Organization in Human EEG

Table S1. Summary Statistics by Dataset

Dataset	N	θ Mean	α Mean	Ratio	r	p-value	φ-org %
PhysioNet	109	6.05 ± 0.31	10.12 ± 0.44	1.68	0.628	2.5e-13	82.6%
LEMON	211	6.05 ± 0.52	9.97 ± 0.85	1.65	0.497	1.4e-14	79.1%
**Combined**	**320**	6.05 ± 0.45	10.02 ± 0.72	1.66	**0.54**	**<1e-20**	**80.0%**

Table S2. Subgroup Analysis (LEMON Dataset)

Subgroup	N	r	p-value	θ Mean	α Mean
Young (<40 y)	142	0.574	8.6e-14	—	—
Old (≥40 y)	69	0.344	3.8e-03	—	—
Male	134	0.429	2.3e-07	—	—
Female	77	**0.680**	1.1e-11	—	—
High φ	105	—	—	6.24	9.75
Low φ	106	—	—	5.85	10.20

Table S3. Monte Carlo Null Model Results

Dataset	Observed r	Null Mean	Null SD	Z-score	Exceeded
PhysioNet	0.628	-0.175	0.093	**8.63**	0/10,000
LEMON	0.497	-0.293	0.061	**12.91**	0/10,000

Note: Null distributions were negative because random within-band frequencies produce anti-correlated PCI and convergence.

Table S4. Epsilon Sensitivity Analysis

ε value	r	p-value	95% CI
0.001	0.41	<1e-10	[0.31, 0.50]
**0.01 (default)**	**0.54**	**<1e-20**	[0.46, 0.62]
0.1	0.62	<1e-25	[0.54, 0.69]
0.5	0.74	<1e-35	[0.68, 0.79]
1.0	0.78	<1e-40	[0.73, 0.82]

Note: Correlations remained positive and significant across all ε values. Effect size increased with larger ε.

Data Availability

Subject-level data for PhysioNet (N=109) are provided in the accompanying Excel file (Supplementary_Data.xlsx). LEMON data are available from the original repository with appropriate data use agreement.

Analysis code is available at: https://github.com/[repository]
